# Observations on the Clinical Features of the Wernicke–Korsakoff Syndrome

**DOI:** 10.3390/jcm12196310

**Published:** 2023-09-30

**Authors:** Michael D. Kopelman

**Affiliations:** King’s College London, Institute of Psychiatry, Psychology & Neuroscience, London SE5 8AF, UK; michael.kopelman@kcl.ac.uk

**Keywords:** Wernicke, Korsakoff, clinical, syndrome, amnesia, memory, confabulation, alcohol, neuropsychology, case reports

## Abstract

This paper begins with a short case report of florid, spontaneous confabulation in a 61-year-old man with an alcohol-induced Wernicke-Korsakoff syndrome. His confabulation extended across episodic and personal semantic memory, as well as orientation in time and place, as measured on Dalla Barba’s Confabulation Battery. Five other brief case summaries will then be presented, followed by a summary of the clinical, neurological, and background neuropsychological findings in three earlier series of Korsakoff patients. These observations will be considered in light of Wijnia’s recent and my own, earlier reviews of the Korsakoff syndrome. Taken together, they indicate the need for a multi-faceted approach (clinical, neurological, neuropsychological, and neuroimaging) to the assessment and diagnosis of the disorder.

## 1. Introduction

Some years ago, my colleagues and I [1] described a patient (AB) whose confabulation extended across episodic, personal, and general semantic memory. The patient herself was a known heavy drinker with a history of previous alcohol-related problems, for which she had been disciplined at work two years earlier. On neurological examination at admission, she showed characteristic features of Wernicke’s encephalopathy (confusion, ataxia, horizontal nystagmus on both left- and right-sided gaze, and ophthalmoplegia with a failure to abduct the eyes in either direction). Despite a course of intravenous thiamine and a later course of intravenous multivitamins, a severe memory deficit emerged when the patient’s global confusion settled. This amnesia was disproportionate to any other cognitive impairment, and characteristic of the Korsakoff syndrome. However, we were struck by the floridity and persistence of this lady’s confabulation over several weeks and, approximately a month after admission, a large necrotic carcinoma of the cervix was diagnosed. An MRI of the head did not show any changes until 5 months after the initial admission, when two posterior cerebral metastases appeared, unaccompanied by any further change in her memory disorder or confabulation. We concluded that ‘non-metastatic’ complications of carcinoma had contributed to the floridity and persistence of the confabulation. Nowadays, an autoimmune complication of the carcinoma would be suspected, and antibodies would have been sought.

In the current paper, I will begin by describing another confabulating Korsakoff patient, seen a few years later, in whom the same neuropsychological assessment was conducted. This patient was not complicated by any underlying carcinoma. The findings will be compared with those in the previous patient. I will then give brief case reports from five other patients and describe summary clinical and neuropsychological findings from my previous studies to make the case for a multi-faceted approach to the assessment and diagnosis of Korsakoff’s syndrome, involving clinical history, neuropsychological testing, physical examination, blood tests, and neuroimaging.

## 2. Case Description

C was a 61-year-old man, who was found collapsed on the pavement by a neighbour. He was unable to give any account of what had happened or his medical history. He was taken to the Accident and Emergency Department at a major London hospital, where he was noted to be confused and disorientated in time and place. He admitted to consuming a bottle of whisky a day. The medical team noted marked nystagmus on left and right lateral gaze. There was mild ataxia on the finger-to-nose test, but he was very ataxic on walking. He could not stand without support. There was no ophthalmoplegia.

Although admitted to hospital during the early 2000s, C gave responses consistent with the 1970s or early 1980s. He said that the month was “December” when it was actually August, and that the year was 1976. He thought that he was aged 42 (he was 61), that Margaret Thatcher was Prime Minister of the U.K., and Richard Nixon was President of the United States. He was also disorientated in place, thinking that he had been admitted to St. Mary’s Hospital, rather than St. Thomas’s Hospital. On crude clinical testing, B’s naming, mental calculation, drawing, and copying appeared to be intact. Nevertheless, he was very obviously memory-impaired and confabulating, believing that he had met various staff members and medical students before, when he had not. A diagnosis of Wernicke–Korsakoff syndrome was made, and C was commenced on a course of high dose multivitamins, administered by intravenous infusion, twice daily for five days.

An MRI brain scan showed widening of the frontal horns of the lateral ventricles, together with enlarged frontal sulci and sylvian fissures bilaterally.

### 2.1. Neuropsychological Testing

#### 2.1.1. Method

C was tested on the Schonell Reading Test [2] to give an estimate of premorbid IQ, and on the Wechsler Abbreviated Scale of Intelligence [3] as an estimate of current IQ. In terms of executive function, C was tested on FAS Verbal Fluency [4], Cognitive Estimates [5], Trail Making A and B [6], and the Modified Card-Sorting Test [7]. For anterograde memory, C was tested on Logical Memory [8], he copied the Rey–Osterrieth Figure, and then was tested on immediate and delayed recall [9]. The Rivermead Behavioural Memory Test [10] was also administered. Autobiographical memory was assessed on the Autobiographical Memory Interview (AMI) [11].

Confabulation was assessed on an English translation [1] of Dalla Barba’s [12,13,14] Confabulation Battery. Results of this battery could be compared with findings previously obtained in patient AB, two non-confabulating patients (D and E), and two age-matched controls [1]. Patient D had bilateral temporal lobe damage, but no frontal damage, and patient E had large infarcts in the medial frontal cortex bilaterally, but no temporal lobe pathology.

#### 2.1.2. Results

Table 1 shows that C’s Full-Scale IQ (90) was relatively preserved, compared with his estimated premorbid IQ (101).

On executive function, he was severely impaired on both FAS Verbal Fluency and the Modified Card-Sorting Test, the latter in terms of both the low number of categories obtained and a high percentage of perseverative errors. His scores at Cognitive Estimates and the Trail Making Test were in the lower range of normal.

C’s scores on standard anterograde memory tests were all severely impaired.

On the Autobiographical Memory Interview (see Table 2), C showed a steep ‘temporal gradient’ in the recall of personal semantic facts, obtaining a maximum score for childhood facts, and a zero score for ‘recent’ facts. In the recall of autobiographical episodes or incidents, C showed a ‘flatter’ gradient with a score of 2 for childhood episodes, 4 for ‘young adult’ life events, and a score of 0.5 for ‘recent’ episodes.

Table 3 shows C’s scores on the Confabulation Battery. He showed confabulation across personal semantic, episodic, and orientation items with a raised score on Don’t Know episodic items, a pattern similar to our previous patient AB [1]. Likewise, the greatest number of confabulations came in the Episodic category. Unlike AB, he did not show a raised occurrence of ‘general semantic’ confabulations.

Examples of C’s confabulations occurred across all categories. He told us that he had just returned from abroad, and that he was living in the hospital because it was his normal accommodation (*personal semantic confabulation*). He said that he had not seen his brother for a long time, because the brother had emigrated—in fact, the brother was visiting C on a weekly basis. He said that he had taken his girlfriend to visit his parents’ house at Christmas and, on another occasion, he told us that he had recently married a local girl in Lancaster—both statements had been true in the distant past, but he was now long separated from his wife (*episodic confabulation*). He thought that the year was 1976, and that he “had been here before” as an aircraft technician (*orientation in time and place*). He thought that the Pope and Marilyn Monroe had both been assassinated—inaccurate, but understandable errors (*general semantic memory*). He said that he had been working as an aircraft technician the previous year—false for that year, but true 30 years earlier *(Don’t Know, episodic),* and that Marilyn Monroe’s father had been a chauffeur (*Don’t Know, semantic*).

At interview, when asked how long he had been in hospital, C said: “I have been here two days [*he had been in hospital approximately 10 weeks at that point*] but on previous projects, I have been here quite a few times when I visited my brother… I am not on any [projects] at the moment, but I have worked with chickens, sorting them out, weighing them and doing a sort of quality control of the beasts.” Asked where he currently was, he claimed he was “on the coast of Essex, inland from the coast of Essex” [*he was in central London*]. Asked about his time in the Air Force 35 years earlier [*remote autobiographical memory]*, C became much more fluent, describing how the chief technician wore a particular arm badge, and he himself was “running an electrical section, providing actuators and all sorts of electrical equipment.” Asked about the Prime Minister or President of Zimbabwe, where he had worked in the distant past, he wondered if it was (Ian) Smith [*from the 1970s*]. Asked who was the current President of the United States, C referred to “that jolly joker, Carter” [*also from the 1970s*]. Likewise, he thought that Margaret Thatcher was still Prime Minister in the U.K. [1979–1990].

### 2.2. Follow-Up and Discussion

C remained with us for approximately 12 weeks until a suitable care home could be found for him. The severity and persistence of C’s confabulation was comparable with that of patient AB (see Table 3), but it occurred in the absence of any complicating illness. His confabulation and its persistence were attributed to the striking degree of focal frontal cortical atrophy on his MRI brain scan, consistent with previous studies which have attributed spontaneous confabulation to frontal pathology, particularly in the ventro-medial and orbito-frontal cortex [15,16,17].

## 3. Some Example Brief Korsakoff Case Reports

There has been recent debate about the diagnosis and clinical features of the Wernicke–Korsakoff syndrome [18,19,20,21,22]. Below are a few case summaries from participants included in my earlier papers [23,24].

F was a 47-year-old female receptionist and secretary. She was being investigated by her GP for possible gastritis. On a domiciliary visit, the GP noted an acute onset of Wernicke features. F was completely disoriented in time and place with bilateral lateral rectus palsies (ophthalmoplegia), which resolved on treatment within a month. The patient was unable to stand, because she was so ataxic. There was what was described as “profuse confabulation” on admission to hospital, but this resolved during the course of the next month. When examined three months later, F showed a few beats of nystagmus on left lateral gaze and marked ataxia on the heel-to-toe walking test. There were complaints of numbness, and a sensory neuropathy was found in her lower legs and feet with impaired proprioception. From informants, we learned that F had consumed three quarters of a bottle of gin plus half a bottle of wine each day for approximately the last 7 years. In the 20 years before that, she was said to have consumed a bottle of sherry and as much as two and a half bottles of wine in a day. Neuropsychological assessment occurred when F had been abstinent for the previous 3 months. On the NART-R [25], F’s score gave an estimated premorbid IQ of 115, and an abbreviated version of the WAIS-R [26] gave a current Full-Scale IQ which was also 115. By contrast, verbal and visual, and recall and recognition anterograde memory tests gave an overall memory index of less than 50.

G was a 62-year-old male schoolteacher. There had been a hospital admission 3 months earlier, when there was no mention of memory symptoms. At the commencement of his current admission, he was diagnosed with Wernicke–Korsakoff syndrome. In the clinical history, there was a record of memory ‘blackouts’, and early morning tremor and nausea. Confabulation had also been noted at admission, but it was not clinically apparent during an assessment after 3 months’ abstinence. He remained completely disorientated in time and place. There was mild bilateral horizontal nystagmus, but no ophthalmoplegia. However, there was marked ataxia on the heel-to-toe test with a wide-based gait. There were absent ankle jerks and an intention tremor on the finger-to-nose test, and also a tremor at rest. There was dysdiadochokinesis (incoordination of movement at the wrists). As far as we could ascertain, G had consumed from 10 to 15 pints of beer daily plus two miniatures of brandy or three to four glasses of gin a day during the previous 20 or more years. At his neuropsychological assessment (when he had been ‘dry’ for 3 months), his estimated premorbid IQ was 110, current Full-Scale IQ 118, and current memory index 65.

H was a 52-year-old male railway and road labourer. Duodenal ulceration had been diagnosed 3 months earlier, and he had been seen at a major hospital for haematemesis (vomiting blood) a month before his current admission. No Wernicke features or memory symptoms were noted during these earlier assessments, and a friend confirmed that he had not been obviously amnesic at these hospital visits. During the current admission, H was found to be disorientated in time with bilateral horizontal nystagmus, but no ophthalmoplegia. He was very ataxic on the heel-to-toe test. There was a sensory neuropathy with absent ankle jerks. Apathy, slowness, lack of initiative, and perseveration were also noted. His liver function tests were abnormal. When assessed 5 months later, H could sometimes give the year and month, but never the precise date, day of the week, or time. From a friend, we obtained a history of his having been a heavy drinker for 30 years or more, consuming at least eight pints of beer in a day during the last 10 years, and latterly a combination of Guinness and cider. He had been abstinent for 5 months at the time of his neuropsychological assessment, when his estimated premorbid IQ was 100, current IQ 102, and current memory index 55.

J was a 59-year-old female worker in the clothing industry. She had been admitted to hospital 8 years earlier, disorientated in time and place with spontaneous confabulation. Ophthalmoplegia, bilateral nystagmus on lateral gaze, a pronounced ataxia, and a peripheral neuropathy with absent ankle and knee jerks, were all noted at admission. When re-assessed 8 years later, she no longer displayed Wernicke features, and she was now abstinent from alcohol. A history was obtained of her consuming from eight to ten glasses of whisky a day for 15 years or more. On neuropsychological testing, her estimated premorbid IQ was 100, with current IQ 100, and a memory index of 50.

K was a 59-year-old male schoolteacher, who was reported to have had an acute onset of Wernicke’s encephalopathy while staying in a hotel in West Africa 7 years earlier. He had suddenly become disorientated in time and place with spontaneous confabulation, severe ataxia, and bilateral nystagmus. He was also described as having been excessively talkative and abusive. Seven years later, he was now partially orientated, not knowing whether it was morning or afternoon, or the day of the week, or the precise date, but able to give the month and year accurately. He was no longer showing spontaneous confabulation. Neurological examination revealed right lateral nystagmus, a few beats of nystagmus on left lateral gaze, and marked ataxia on the heel-to-toe test. He admitted to heavy drinking in the immediate years pre-hospital admission, and a past history had been recorded of his consuming from eight to nine pints of beer at night plus a third to a half bottle of spirits daily. He had served in the Fleet Air Arm during his 20s, then the Merchant Navy, but he had not been able to remember his career details after that, suggesting that his heavy drinking and memory problems extended back 30 years or more. He himself stated, and his care staff confirmed, that his drinking was now very well controlled; he consumed only about a pint of beer a day. On neuropsychological testing, K had an estimated premorbid IQ of 124, a current Full-Scale IQ of 129, and a current memory index of 69.

## 4. Summarised Clinical Findings in Previous Korsakoff Studies

Kopelman [23] recruited 16 such alcoholic Korsakoff patients, selected to conform as closely as possible to the clinical features of an ‘acute onset’ subgroup identified by Cutting [27] in a previous (retrospective) study of Maudsley Hospital patients (defined as less than 8 weeks between onset of symptoms and admission). These patients had a similar sex ratio (13 male: 3 female) and mean age (56.5, range 38 to 66) to Cutting’s group. All had a history of very heavy and prolonged alcohol misuse. Ten of the 13 male participants had served in the Navy, which is where their heavy drinking had often commenced. The mean length of drinking history was 24 years (range 10 to 37.5) and median time ‘dry’ was 24 weeks (range 8 weeks to 12 years). With respect to Wernicke features, all cases had a history of disorientation in time, and all had a history of ataxia; 12 out of 16 cases had a recorded history of nystagmus and/or ophthalmoplegia, and 10 cases also had a history of peripheral neuropathy. Although some patients had shown a degree of improvement on repeated psychometric testing, all were severely incapacitated by their memory disorder, either living in institutions or heavily dependent on carers’ or institutional support. The mean estimated premorbid IQ was 106.3 (±11.5) with a mean Full-Scale IQ of 101.8 (±12.3) and a mean memory index of 77.4 (±7.3).

Kopelman [24] included 16 Korsakoff patients, who had attended psychiatric hospitals around London. The patients had a sex ratio of 11 men to 5 women and a mean age of 53.6 (range 38 to 65). The rate of onset had been ‘acute’ in all cases for whom precise details were available. All patients had a history of very heavy and prolonged alcohol abuse (the mean length of drinking history was 23.0 years, and the range from 13 to 37.5 years), while the mean time ‘dry’ was 5.75 years (the range was from 1 to 15 years). With respect to Wernicke features, all cases had a recorded history or current evidence of disorientation in time; 14 had been ataxic, 11 cases had shown nystagmus and/or ophthalmoplegia, and 10 an associated peripheral neuropathy. All patients were either living in institutions or heavily dependent on carers’ or institutional support. A clinical memory and orientation scale confirmed severe memory impairment. The mean estimated premorbid IQ was 104.3 (±10.6) with a current Full-Scale IQ of 105.25 (±10.7) and a mean memory index of 54.7 (±10.65). All patients showed a mild degree of cortical atrophy on a CT scan with variable severity of frontal lobe atrophy [24].

Kopelman et al. [28] recruited 13 patients with an alcoholic Korsakoff syndrome; 12 out of these 13 patients had either a documented history or residual signs of a Wernicke episode preceding their amnesic syndrome. In all 13 patients, memory was affected out of all proportion to other cognitive deficits. The mean age of the Korsakoff patients was 55.4 years (range from 43 to 68), and the sex ratio was ten men to three women. The mean length of drinking history was 27.5 years (range from 18 to 48 years), and the mean time ‘dry’ was 3.50 years (range from 2 months to 17 years). The mean estimated premorbid IQ was 107.8 (±12.5), the current Full-Scale IQ = 97.4 (±14.2), with a WMS-R General Memory Index of 66.6 (±19.9) and Delayed Memory Index of 59.3 (±15) [29]. Quantitative MRI and PET scan investigations were carried out and the findings were reported elsewhere [30].

## 5. Discussion

If the Korsakoff syndrome is defined as “an abnormal mental state in which memory and learning are affected out of all proportion to other cognitive functions in an otherwise alert and responsive patient, resulting from nutritional, i.e., thiamine, depletion” [19,31], it is certainly possible to find such patients, as corroborated by the above observations. Debate has revolved around the question of whether or not the diagnosis should be broadened beyond this definition to incorporate patients with more widespread cognitive impairments [18,32,33,34]. Elsewhere, I have argued that it is neither necessary nor desirable to broaden this definition [19], and I do not propose to reiterate those arguments here.

Following the publication of my paper on the definition of the disorder [19], Wijnia [35] published an excellent review of the clinical features of the Wernicke–Korsakoff syndrome, and it may be useful to compare the above findings with remarks made in Wijnia’s paper [35], as well as my own earlier reviews [19,31,36,37]. Unlike those authors who have sought to re-write or expand the definition of the Korsakoff syndrome, Wijnia [35] sought, more modestly, “to provide a clinical framework for (timely) diagnosis of Wernicke’s encephalopathy in order to prevent the permanent brain damage associated with Korsakoff syndrome”.

Wernicke’s syndrome (or encephalopathy) is indeed commonly missed. Wernicke [38] described confusion, ataxia, ophthalmoplegia, and nystagmus in two alcoholic patients and one patient with pyloric stenosis [31]. Korsakoff [39] also mentioned in passing “prodromal agitation and confusion”, ophthalmoplegia, nystagmus, and ataxia, without attributing them to Wernicke’s earlier description of the syndrome (see also [40,41]). Subsequent studies have given widely varying prevalence rates for these features preceding the Korsakoff syndrome [27,42,43,44]. Kopelman [19] and Wijnia [35] both described a broad range of possible alternative or confounding diagnoses that can cause confusion (delirium) in alcoholic misuse disorder. Other studies [27,31,36,45,46,47,48] have reported that the initial manifestations of the Korsakoff syndrome may vary from acute coma or confusion (delirium, which may sometimes include transient psychotic-like features), through the classical Wernicke syndrome, to an insidious onset of memory impairment and, in some cases, the disorder may not be identified until the patient comes to autopsy [31,36,45,46,47,49].

In my opinion, there is a lot to be said for simply describing the presence or absence of the four main Wernicke features (as in the case reports and summarised clinical findings provided in the preceding two sections of this paper), but this is not often done (in part, perhaps, because these features are sometimes poorly documented in the medical records). Instead, Caine et al. [21] have attempted to operationalise diagnostic criteria for Wernicke’s syndrome. However, they included ‘dietary deficiencies’ and ‘mild memory impairment’ among their diagnostic criteria, thus differing “substantially from the classic definition” (their words) [21]. They found that the overall diagnostic sensitivity of Wernicke’s encephalopathy was increased from 23% (using the traditional ‘triad’ of confusion, ataxia, and ‘eye signs’) to 85% (using any two of their revised criteria) [21,35], and their method has been used with success by others [50].

Many previous studies have reported that Wernicke signs can follow self-starvation, intravenous feeding (especially in the presence of a glucose load), the persistent vomiting of hyperemesis gravidarum, and carcinomas of the gastrointestinal tract [31,51,52]. Ebels [53] stated that the underlying clinical disorders, where alcohol misuse has been excluded, fall into four main groups: (i) gynaecological (hyperemesis gravidarum, malignancy); (ii) gastrointestinal (carcinoma, other causes of malabsorption); (iii) other malignancy; and (iv) other causes of debility and malnutrition (e.g., haemodialysis, severe self-neglect, severe or chronic infection) [31,53]. In their study, Wijnia et al. [54] found that concurrent infections were reported in 51% of patients in the initial Wernicke phase, including pneumonia, urinary tract infections, abscesses, empyema, and sepsis without a known source. Such infections may be the precipitant of Wernicke features in patients with marginal thiamine reserves [55], but they can also be a complicating factor of the thiamine deficiency itself. Oudman et al. [52] and Wijnia et al. [56] also reported an association with malignancies, which were found in approximately 22% of non-alcoholic Wernicke cases [52] and inpatient Korsakoff cases [56] (compare [1,31]).

Wijnia [35] found that vomiting, weight loss, and visual complaints occur more frequently in non-alcohol-related Wernicke encephalopathy than in the alcohol-induced syndrome. However, these non-alcoholic patients are less susceptible to chronic confusion [35] and prolonged cognitive deficits than alcoholics, which Kopelman [31] attributed to generally higher standards of premorbid nourishment, and very likely more prompt treatment. Consistent with this, Oudman et al. [57] reported an excellent outcome in a Wernicke case following bariatric surgery, complicated by COVID-19, in whom high-dose thiamine intravenous treatment was delayed but then administered for over 2 months.

Cholinergic depletion may also contribute to the pathogenesis of cognitive and autonomic dysfunction in the Wernicke–Korsakoff syndrome, as thiamine appears to be involved in cholinergic synaptic transmission, and its depletion may cause decreased bioavailability of acetylcholine [35]. Similar arguments were put forward in the 1980s by Witt [58], who argued that six neurotransmitter systems (including acetylcholine, GABA, and glutamate) were affected by thiamine depletion, either by reduction in thiamine pyrophosphate-dependent enzyme activity or by direct structural damage. Arendt et al. [59] reported a 47% reduction of the neuron count in the nucleus basalis of three Korsakoff patients at autopsy. Kopelman and Corn [60] showed that cholinergic depletion produced an anterograde amnesia, similar to that seen in the Korsakoff syndrome, but could not account for the retrograde memory loss seen in Korsakoff’s syndrome.

Only a minority of people with alcoholic misuse/dependency develop the Wernicke–Korsakoff syndrome, and a number of studies have sought to identify a genetic predisposition for developing the syndrome [61,62,63]. Blass and Gibson [61] proposed a hereditary abnormality of transketolase metabolism. Guerrini et al. [62] obtained abnormalities on SLC19A2 and SLC19A3, which they proposed affected the pathophysiology of thiamine deficiency. More recently, Jimoh et al. [63] identified an m.A3243G mtDNA mutation, associated with mitochondrial dysfunction, in a single case of a young woman with Wernicke–Korsakoff signs and the apparent subsequent development of schizophrenia. The latter was a single case report only, and the diagnosis of schizophrenia, as opposed to an affective or some other psychosis, remains somewhat uncertain.

The pattern of anterograde and retrograde memory loss in the Korsakoff syndrome has been reviewed in many places elsewhere, e.g., [18,31,36,64,65,66]. There are particular deficits in context memory (especially for temporal order) [24,36,67,68,69,70,71,72,73,74,75,76,77,78,79,80]. The retrograde amnesia usually involves a temporally-graded memory loss, extending back 25 years or more [24,28,81,82,83,84]. The specific neuroimaging findings characteristically associated with the Korsakoff syndrome have also been reviewed elsewhere [36] with characteristic atrophy on quantified structural MRI in the thalami and mammillary bodies, often associated with frontal lobe volume loss [85,86,87], and changes in glucose metabolism on FDG (fluoro-deoxy-glucose) PET (positron emission tomography) in the thalamus and associated limbic circuitry [30].

In the present paper, spontaneous confabulation was found in patient C, extending across episodic memory, orientation in time and place, and personal semantic memory, as had been found in patient AB before him [1], and these confabulations were most common in episodic (autobiographical) memory. C’s underlying pathophysiology was not complicated by any additional diagnosis (unlike AB, in whom carcinoma was found with probable autoimmune effects). Like AB, the confabulations persisted for at least 12 weeks; almost certainly the consequence of B’s severe bilateral frontal lobe atrophy, involving the ventro-medial and orbito-frontal brain regions [16,17,88,89,90,91,92]. The usual pattern in spontaneous confabulation is that it subsides for over the course of between 9 months and 3 years following onset [93,94] (consistent with cases J and K, above) or sooner (cases F and G, above). Recent studies, using objective tests of confabulation, have found persisting confabulation in at least some Korsakoff patients [95,96]. Kopelman [97] found persisting ‘momentary’ confabulation in 50% of Korsakoff cases, although not persisting spontaneous confabulation. Wijnia [35] concluded that “patients with Korsakoff syndrome can exhibit confabulations, although the intensity and frequency can vary” across patients.

With respect to the course of the disorder, and its associated morbidity and mortality, Victor et al. [44] and Kopelman et al. [37] argued that as many as 75% of cases show some degree of improvement through time, provided that they are adequately treated with high-dose thiamine at admission and then manage to refrain from further alcohol misuse, which many patients fail to do once returned to the community. Interestingly, patient K (above) successfully moderated his alcohol consumption to one pint of beer daily after many years of heavy drinking. Wijnia [35] argued that the outcome can be very variable following a Wernicke episode, suggesting that there can be recovery, a persistent Korsakoff syndrome, or continued decline, depending on whether the initial encephalopathic stage has resulted in lasting damage. Although ataxia generally resolves 3–6 months following onset, in some cases it can be severe and persistent [37], and result in the patient becoming wheelchair-dependent [35]. Wijnia [35] suggested that the neuropsychological assessment of Korsakoff syndrome can commence after at least 6 weeks of alcohol abstinence, but this period may be even longer if delirium persists. My own experience suggests that it often takes 3 months before the patient becomes testable and the prognosis starts to clarify; see also [37]. In chronic patients in residential care, associated disorders include cirrhosis, chronic obstructive pulmonary disease, peripheral artery disease, stroke, diabetes, and epilepsy, some of which may also be related to a history of heavy smoking [19,35]. Wijnia [35] reported that the median survival in Korsakoff syndrome was 10.7 years [98], and that mortality resulted from vascular pathology, infections, gastro-intestinal diseases, head injury and other accidents, and suicides.

## 6. Summary and Conclusions

While there is no need to change or broaden the core definition of Korsakoff’s syndrome itself [19], there is wide variability in the associated features of the Wernicke–Korsakoff syndrome. Wernicke’s encephalopathy is commonly missed and, while Caine et al.’s [21] operational criteria can increase the identification of the syndrome, there is also something to be said for carefully recording and reporting the four key features of the Wernicke syndrome (which is not often done). In alcohol-induced Korsakoff’s syndrome, the initial manifestations of the disorder may vary from acute coma, confusion, through the classical Wernicke features, to an insidious onset of memory impairment. In some cases, the disorder may not be identified until the patient comes to autopsy. In non-alcohol-induced Wernicke’s encephalopathy, there can be a wide variety of precipitating disorders, but they usually fall into four main groups: (i) gynaecological (hyperemesis gravidarum, malignancy), (ii) gastrointestinal (carcinoma, other causes of malabsorption), (iii) other malignancy, and (iv) other causes of debility and malnutrition (e.g., infection, haemodialysis, severe self-neglect). Taken together, these various factors indicate that the assessment and clinical diagnosis of Wernicke–Korsakoff syndrome requires a multi-faceted approach, incorporating a detailed clinical history (often from an informant, if available), physical (including neurological) examination, blood tests, neuropsychological testing, and neuroimaging.

Spontaneous confabulation may extend across all aspects of memory, including orientation in time and place, episodic and personal semantic memory (as in Patient C), and general semantic memory (as in AB). It occurs most commonly in episodic/autobiographical memory. It tends to subside through time, leaving just a residuum of ‘provoked’ or momentary confabulations, but some recent studies have pointed to a persistence of spontaneous confabulation in at least some cases.

The long-term course of Korsakoff’s syndrome is very variable but, in the absence of malignancy, the prognosis in Wernicke’s encephalopathy is generally better in non-alcoholic than alcoholic cases, which is very likely the consequence of better premorbid health and prompter treatment. In alcohol-related Wernicke–Korsakoff cases, it may take from 6 to 12 weeks from hospital admission for a settled pattern of neuropsychological performance to emerge, and for the likely prognosis to become evident. Outcome may depend in large part on whether the patient resumes heavy drinking, but other life habits, such as cigarette smoking and substance misuse, and associated disorders, including head injury, liver disease, and vascular pathology, are often the ultimate determinants of life expectancy.

Thiamine depletion alone does not always produce Wernicke–Korsakoff pathology [99], and depletion of other B vitamins can contribute to the cognitive impairment [100]. At a basic science level, future research needs to examine how thiamine depletion interacts with genetic predisposition and other neurochemical or vitamin depletions to produce the syndrome. At a clinical and neuropsychological level, future studies need to separate out the core Korsakoff features from those of the many other alcohol-induced syndromes [19,35] to elucidate how underlying brain pathophysiology gives rise to these other syndromes, and whether and how these other pathophysiologies interact with the Wernicke–Korsakoff pathology.

## Figures and Tables

**Table 1:** C’s performance on IQ, executive, and anterograde memory tests.

*Estimated Premorbid IQ*:	(Schonell)	101
*Current (WASI) IQ:*	Verbal	95
	Performance	85
	Full Scale	90
*Executive tests:*		
FAS	9 (severely impaired)	
Cognitive estimates	8 (borderline)	
Trails A	<25th percentile	
Trails B	<25th percentile	
Modified Card-Sorting:		
Categories	1/6	
% perseverative errors	75%	
*Anterograde memory:*		
Logical memory	immediate:	2nd percentile
	delayed:	1st percentile
Rey figure	copy:	50th percentile
	immediate recall	1
	delayed recall:	0
RBMT	picture recognition	Chance

**Table 2:** C’s performance on the Autobiographical Memory Interview.

	‘Personal Semantic’ \Facts (Max = 21)	Autobiographical Incidents (Max = 9)
Childhood:	21	2
Young adult:	7	4
Recent:	0	0.5

**Table 3:** Performance of patients C and AB on Dalla Barba’s Confabulation Battery ^1^.

Confabulation Battery	Patient C	Patient AB	Patient D (Temporal)	Patient E (Frontal)	Control (Means)
Personal Semantic	7	4.8	0	7	0
Episodic	11	15.3	0	0	0
Orientation	9	4.8	0	0	0
General Semantic	2	7.8	1	0	5.0
DK Semantic	1	1.3	0	2	2.0
DK Episodic	5	7.5	0	5	2.0

^1^ This table contains some data originally described in Reference [1], although not previously presented in this format.

## Data Availability

No new data were created. The empirical investigations cited were conducted in the 1980s and 1990s with ethical approval, and the data were not archived.
